# Stable Isotope Tracing Uncovers Reduced γ/β-ATP Turnover and Metabolic Flux Through Mitochondrial-Linked Phosphotransfer Circuits in Aggressive Breast Cancer Cells

**DOI:** 10.3389/fonc.2022.892195

**Published:** 2022-05-31

**Authors:** Aleksandr Klepinin, Sten Miller, Indrek Reile, Marju Puurand, Egle Rebane-Klemm, Ljudmila Klepinina, Heiki Vija, Song Zhang, Andre Terzic, Petras Dzeja, Tuuli Kaambre

**Affiliations:** ^1^Laboratory of Chemical Biology, National Institute of Chemical Physics and Biophysics, Tallinn, Estonia; ^2^Department of Cardiovascular Medicine and Center for Regenerative Medicine, Mayo Clinic, Rochester, MN, United States; ^3^Department of Chemistry and Biotechnology, School of Science, Tallinn University of Technology, Tallinn, Estonia; ^4^Laboratory of Chemical Physics, National Institute of Chemical Physics and Biophysics, Tallinn, Estonia; ^5^Laboratory of Environmental Toxicology, National Institute of Chemical Physics and Biophysics, Tallinn, Estonia; ^6^Department of Molecular Pharmacology and Experimental Therapeutics, Mayo Clinic, Rochester, MN, United States; ^7^Department of Clinical Genomics, Mayo Clinic, Rochester, MN, United States

**Keywords:** triple-negative breast cancer, 18 O stable isotope labeling technology, γ-and β-ATP phosphoryl turnover, adenylate kinase, creatine kinase, oxidative phosphorylation, glycolysis, phosphotransfer network

## Abstract

Changes in dynamics of ATP γ- and β-phosphoryl turnover and metabolic flux through phosphotransfer pathways in cancer cells are still unknown. Using ^18^O phosphometabolite tagging technology, we have discovered phosphotransfer dynamics in three breast cancer cell lines: MCF7 (non-aggressive), MDA-MB-231 (aggressive), and MCF10A (control). Contrary to high intracellular ATP levels, the ^18^O labeling method revealed a decreased γ- and β-ATP turnover in both breast cancer cells, compared to control. Lower β-ATP[^18^O] turnover indicates decreased adenylate kinase (AK) flux. Aggressive cancer cells had also reduced fluxes through hexokinase (HK) G-6-P[^18^O], creatine kinase (CK) [CrP[^18^O], and mitochondrial G-3-P[^18^O] substrate shuttle. Decreased CK metabolic flux was linked to the downregulation of mitochondrial MTCK1A in breast cancer cells. Despite the decreased overall phosphoryl flux, overexpression of HK2, AK2, and AK6 isoforms within cell compartments could promote aggressive breast cancer growth.

## 1 Introduction

One of the cancer hallmarks is the ability to reprogram energy metabolism to support malignant cell proliferation and metastatic activity (1, 2). However, despite significant advances, the molecular mechanisms and logic of such metabolic rearrangements and adjustments are still a mystery (3). It is unknown how mitochondrial ATP production is connected with sites of ATP utilization in different cellular compartments to support the growth of malignant cells. Unveiling ATP production and ATP consumption dynamics in cancer cells will facilitate the development of a new therapeutic strategy for human breast cancer (HBC), the most diagnosed tumor and the second cause of death among females worldwide (4). Recently, the hybrid glycolysis/oxidative phosphorylation (OXPHOS) metabolic model for aggressive Triple-negative breast cancer (TNBC) cells has been proposed (5). The metabolic hybrid cancer phenotype is one of the reasons why malignant cells incline to metastasis and are cancer therapy-resistant (6). Understanding how OXPHOS interacts with glycolysis will give us new insights into the metabolic plasticity of breast cancer cells.

In cellular energetics, mitochondria are integrated with specialized phosphotransfer circuits that distribute energy from mitochondria and deliver it to ATP consumption sites to support constant undisturbed metabolic homeostasis (7–9). ATP delivery phosphotransfer circuits are mainly comprised of creatine kinase (CK), adenylate kinase (AK), and glycolytic/glucogenolytic enzymes, along with the associated mitochondrial substrate shuttles such as glycerol-3-phosphate (G-3-P) dehydrogenase/glycerol kinase (GPDH/GK) (9–11).

The enzyme AK, which catalyzes the reaction 2ADP ⇆ AMP+ATP, is the central mediator of intracellular nucleotide exchange and AMP metabolic signaling (12). Importantly, AK can deliver both γ- and β-phosphoryl groups of ATP and make them available for utilization, thus doubling the ATP energetic potential (8). Evidence is accumulating that different AK isoforms are involved in cancer development and cell proliferation (13–15). However, it is unknown how changes in the AK-mediated β-ATP turnover and phosphotransfer flux within cell compartments are related to the aggressiveness of the TNBC subtype.

Glycolytic enzymes distributed throughout the cell can also comprise a phosphotransfer network. Hexokinases 1 and 2 (HK1 & HK2), enzymes catalyzing the first step of glycolysis, can relay mitochondrially generated ATP to energy-consumption sites (11, 16). Upregulation of HK2, which has a higher affinity to mitochondria, has been associated with enhanced aerobic glycolysis and promotion of tumor growth in many types of cancers, including HBC (17, 18). It is still unknown how the HK expression pattern correlates with glucose-6-phosphate (G-6-P) turnover and phosphoryl flux through the glycolytic pathway. Such data should provide valuable information about the integration of glycolytic phosphotransfer network into the bioenergetics of HBC.

Another phosphotransfer enzyme, mitochondrial creatine kinase MtCK, which is localized in the mitochondrial inner membrane compartment, facilitates phosphate transfer from mitochondrial ATP to the phosphocreatine (PCr). PCr serves as a dynamic cellular high-energy phosphoryl distributor and buffers during increased energy demand (7, 10). In the epithelial cells, the ubiquitous uMtCK (gene MtCK1) is co-expressed with the cytosolic brain-type CK (B-CK, gene CKB) (7), which is usually up-regulated during malignant epithelial transformation (19). Recent *in vivo* and *in vitro* studies demonstrate that CK’s role in breast cancer progression depends on HBC subtypes (20). However, it is unknown how remodeling of the CK isoform network affects the CrP metabolic flux and energetics of HBC and aggressiveness of TNBC cells.

^18^O is a natural, stable and non-radioactive isotope of oxygen with a natural abundance of 0.2% (21) and more importantly it is not toxic for humans (22). In the cells the newly developed ^18^O isotope-based metabolite tagging technology allows monitoring phosphotransfer dynamics by simultaneously determining their intracellular phosphometabolite levels and turnover rates (23, 24). In metabolically active cells, ^18^O atoms from ${\text{H}}_{2}^{18}\text{O}$ incorporate into phosphoryl groups during each event of ATP hydrolysis. Next, within the cellular phosphotransfer network, the ^18^O-labeled phosphoryls are distributed among phosphate-carrying molecules (23). The resulting ^18^O-labeled phosphates can be detected by gas chromatography-mass spectrometry (GC-MS) or ^18^O-assisted ^31^P NMR, which allows simultaneous detection labeling of G-6-P, G-3-P, PCr, ATP, and other phosphate metabolites (23, 24).

The aim of this study was to adapt ^18^O stable isotope labeling technology for cancer cell cultures to discover how changes in ATP γ- and β-phosphoryl turnover and energy distribution between phosphotransfer pathways affects cancer cell aggressiveness. The current study demonstrates that the ^18^O-based metabolite tagging technology revealed simultaneous depression of rates of ATP synthesis as well as AK, CK, and HK catalyzed phosphotransfer, together with the activity of mitochondrial substrate G-3-P shuttle in aggressive cancer MDA-MB-231 cells representing TNBC. Decreased CK metabolic flux and downregulation of mitochondrial MTCK1A in breast cancer cells indicate rewiring of phosphotransfer circuits. Discovered overexpression of HK2, AK2, and AK6 isoforms in mitochondrial and nuclear compartments suggests that aggressive cancer cells use a strategy of microcompartmentation to support specific cellular functions and promote tumor growth. This study uncovers new features of cancer cell energetics and provides unique insights into the remodeling of the phosphotransfer enzyme network in breast cancer cells that could be used to facilitate the development of new treatment strategies for TNBC.

## 2 Materials and Methods

### 2.1 Chemicals, Kits and Reagents

All cell lines were obtained from the American Type Culture Collection and were maintained under conditions described by the manufacturer: MCF7 (ATCC, HTB-22), MDA-MB-231 (ATCC, HTB26), MCF10A (ATCC, CRL-10317). MEGM Single Quots Supplements (CC-3151) were purchased from Lonza (Switzerland). Dulbecco’s modified Eagle’s medium (DMEM), heat inactivated horse serum and human re-combinant Zn insulin were purchased from Gibco (UK). Sodium pyruvate and fetal bovine serum (FBS) were purchased from Corning (USA). Trizol reagent solution was purchased from Life Technologies ((Thermo Fisher Scientific, USA). The RNeasy Mini kit was obtained from QIAGEN Sciences (Germany). The High Capacity cDNA Reverse Transcription Kit with RNase Inhibitor was acquired from Applied Biosystems (Thermo Fisher Scientific, USA). ${\text{H}}_{2}^{18}\text{O}$ water was received as a Cambridge Isotope Laboratories, Inc Research Award donation from the Cambridge Isotope Laboratories, Inc Sodium pyruvate were purchased from Capricorn Scientific GmbH. Creatine kinase (rabbit muscle) was purchased from Roche Diagnostics GmbH (Mannheim, Germany). The myokinase (rabbit muscle), glycerol kinase (Baccillus stearothermophilus), methanol, glycerol and cholera toxin from Vibrio cholerae were purchased from Sigma Aldrich (USA). Tetra-n-butylammonium hydrogen sulfate (TBHS) was purchased from Acros Organics (New Jersey, USA), MOX (n-Methoxyamine) and MSTFA+1%TMCS derivatization reagents and Pierce BCA Protein Kit from Thermo Fisher Scientific (USA).

### 2.2 Isolation of RNA, cDNA Synthesis, and qPCR

The expression pattern of phosphotransfer network was determined by analyzing the mRNA level. The total RNA from ~10^6^ cells MCF10A, MCF7 and MDA-MB-231 was isolated using the Trizol reagent. RNA purification was performed by RNeasy Mini Kit with DNase treatment. RNA purity at A_260/280_ ratio (range 2.0-2.1) and concentration at 260 nm were measured by a Nanodrop spectrophotometer. 350 ng of total RNA was used for cDNA synthesis, cDNA was synthesized using a High Capacity cDNA Reverse Transcription Kit with RNase Inhibitor. Synthesized cDNA was subjected to quantitative PCR analysis using the TaqMan^®^ Gene Expression Master Mix and FAM-labelled specific primers (Applied Biosystems/Thermo Fisher Scientific, USA): actin beta – Hs01060665_g1; hexokinase I – Hs00175976_m1; hexokinase II – Hs00606086_m1; creatine kinase brain-type HS00176484_m1; creatine kinase, mitochondrial 1B – Hs00179727_m1; adenylate kinase 1 HS00176119_m1; adenylate kinase 2 HS01123132_g1; adenylate kinase 4 HS03405743_g1. All qPCR experiments were performed on a LightCycler 480 II Real-Time PCR System (Roche, Basel, Switzerland). Reactions were carried out in four replicates for each of three independent experiments. Threshold cycles (Ct) were automatically calculated by the LightCycler 480 software (Roche, Basel, Switzerland). Data were analyzed with the formula 2–ΔΔCt (25), normalized to the endogenous control ActB and expressed as fold change over MCF10A samples.

### 2.3 Immunoblotting

Cells were washed twice with Ca/Mg-free PBS and then treated with a Tris-Triton X lysis buffer consisting of: 10 mM Tris pH 7.4, 100 mM NaCl, 1 mM EDTA, 1% Triton X-100, 10% glycerol, 0,1% SDS) supplemented with protease inhibitor cocktail (Roche). Lysates were homogenized by a Retsch Mixer Mill at 25 Hz for 2 min, incubated for 20 min on ice, and clarified by centrifugation at 21,000 g for 30 min at 4°C. The concentration of the isolated proteins is determined using the Pierce™ BCA Protein Assay Kit (Thermo Scientific, Rockford, U.S.A). Protein samples (35 µg) were separated by a 12% Tris-Glycine SDS-PAGE and electrophoretically transferred onto Immobilon® -P PVDF membrane pore size 45 µm (Merck Millipore, Tullagreen, Ireland) by Trans- Blot Semi-Dry Transfer system (Bio-Rad). Membranes were then incubated with the primary antibodies against AK2 (sc374095) or AK6 (10544-1-AP, Proteintech) and α-tubulin (ab7291) and the HRP conjugated secondary antibodies Goat Anti-Mouse IG (ab97040) and Goat Anti-Mouse IG (ab6721). Mouse kidney homogenate was used as a positive control for AK2. MCF7 serves as a positive control for AK6 recommended by the antibody manufacturer. Blots were developed using SuperSignal™ West Femto Maximum Sensitivity Substrate (Thermo Scientific, Rockford, U.S.A.) and imaged with Biospectrum Multispectral imaging system (Biosoectrum 510, UVP, Cambridge, UK).

### 2.4 Phosphometabolomics Analysis

**Figure 1:**
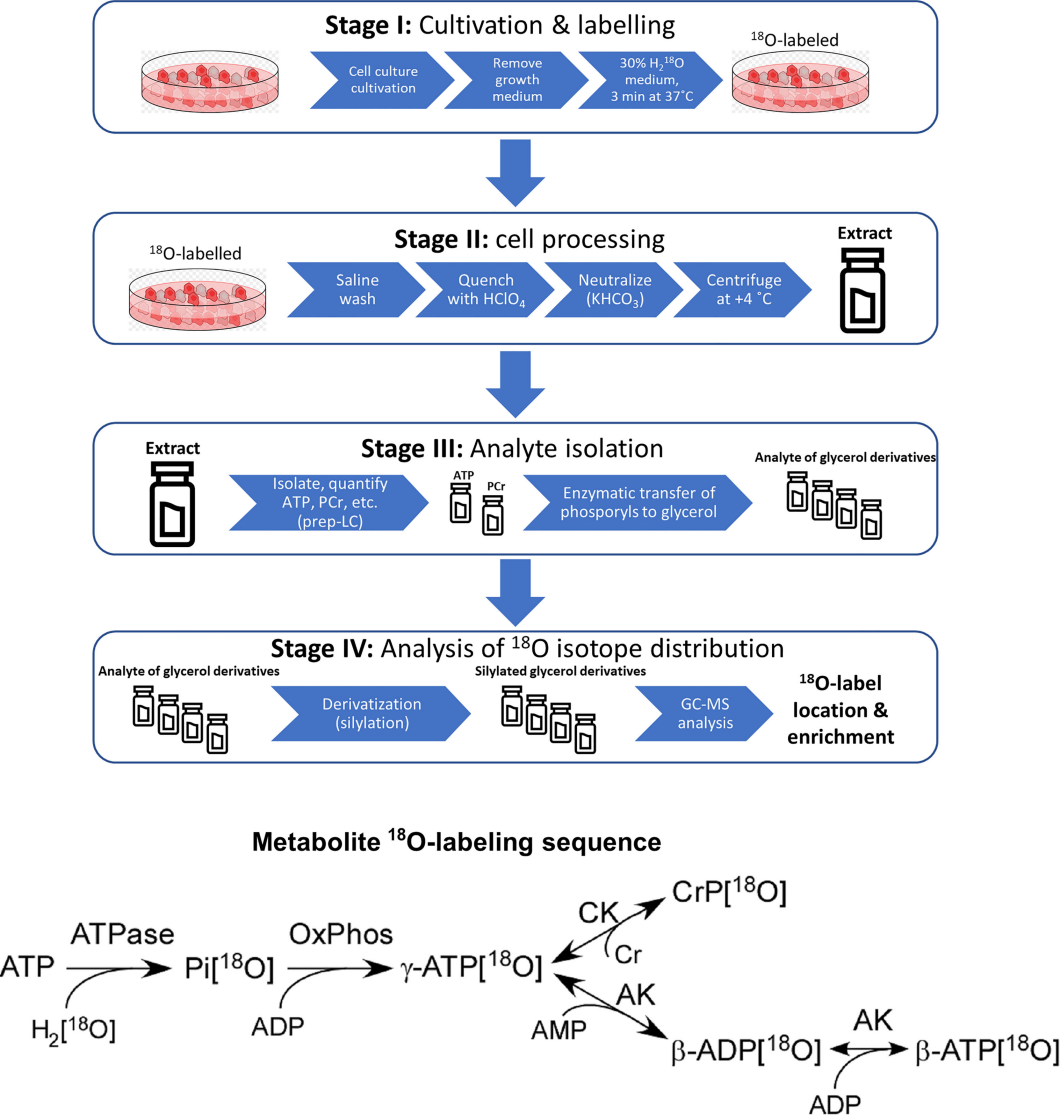
Sample preparation workflow for ^18^O-labelling based phosphometabolomics and metabolite ^18^O-labeling sequence. AK, adenylate kinase; CK, creatine kinase; Cr, creatine, CrP, creatine phosphate; Pi, inorganic phosphate; OxPhos, oxidative phosphorylation; prep-LC, preparative liquid chromatography; GC-MS, gas chromatography-mass spectrometry.

Experimental work towards analyzing the phosphometabolomic flux through energy production pathways was divided into 4 distinct stages (Stage I – Stage IV). The general workflow is depicted in **Figure 1** and described in technical detail further below.

#### 2.4.1 Stage I: Cell Culturing and Labeling

##### 2.4.1.1 Cell Culturing

All cells were cultivated at 37°C in 100 mm (seeding density 2.2x10^6^ cells) or 35 mm (seeding density 0.3x10^6^ cells) Petri dishes in a humidified incubator containing 5% CO_2_ in air and were cultured at 5 days (MCF10A) or 2-3 days (MDA-MB-231 and MCF7) intervals. MDA-MB-231 and MCF7 cells were grown as adherent monolayers in high glucose (25 mM) Dulbecco’s modified Eagle’s medium (DMEM) with stable L-glutamine and sodium pyruvate, supplemented with 10% heat-inactivated fetal bovine serum and 10 μg/mL human recombinant Zn insulin. MCF10A cells were grown in a lower level of glucose (8 mM) in mammary epithelial growth medium (MEGM) supplemented with MEGM SingleQuots Supplements, 5% horse serum and cholera toxin (100 ng/mL). All cell lines were cultured under conditions described by the manufacturer.

##### 2.4.1.2 ^18^O Labeling Procedure for Cell Lines

Cultured cells were labelled with ^18^O molecule ( ${\text{H}}_{2}^{18}\text{O}$ as a stable isotope source) followed a protocol previously described for establishing the energetic profile of muscle cells (24). After reaching 70% of confluency, medium from cultured cells was removed and replaced with regular medium (DMEM for MCF7 and MDA-MB-231 cells and MEGM for MCF10A cells (see *Cell Culturing* section)) enriched with 30% of ${\text{H}}_{2}^{18}\text{O}$ , then cells were incubated for 3 min at 37°C. Cells not treated with ${\text{H}}_{2}^{18}\text{O}$ served as the reference unlabeled background control samples.

#### 2.4.2 Stage II: Cell Processing

The ^18^O labeling process was terminated by rapid removal of the growth medium, washing cells with saline, and quenching with 0.6 M ice-cold HClO_4_ or ice-cold methanol-water (1:1, v:v) (100 µL for 35 mm dish and 300 µL for 100 mm dish). Petri dishes with cells were immediately frozen in liquid nitrogen to quench the cells’ metabolism. Cells were scraped from the Petri dishes with a liquid nitrogen cooled spatula and transferred into cold microcentrifuge tubes, which were immediately placed into liquid nitrogen. Samples were briefly allowed to warm until first signs of thawing could be observed, then centrifuged at 4°C at 10,000 g for 5 min. The supernatant of methanol-water quenched cells was immediately placed into liquid nitrogen, and samples were stored at –80°C until they were analyzed by GC-MS. The supernatant of HClO_4_ quenched cells was removed and transferred to a microcentrifuge tube containing 2 M KHCO_3_ 35 µL for 35 mm dish and 105 µL for 100 mm dish to neutralize the acid and adjust pH to approximately 7.4. The resulting suspensions were centrifuged at 10,000 g for 15 min at 4°C to remove the salt. The supernatant was adjusted to 1 mL with ultrapure water and stored at –80°C until analysis. The remaining pellets were stored in 300 µL of 1%SDS containing 0.1 M NaOH at -20 °C for protein assay with Pierce BCA Protein Kit.

#### 2.4.3 Stage III: Analyte Quantification and Isolation

##### 2.4.3.1 Analyte Quantification

50 µL of the sample was used for phophometabolite quantification for 100mm dish and 20 µL for 35mm dish. ATP and PCr levels and ATP/ADP ratios were determined by a UPLC method adapted and modified from (15, 26). Analytes were resolved on a reversed-phase C18 column (Separon SGX 5 µm 3x150 mm, Tessek, Czech Republic) on a Waters Acquity UPLC equipped with a PDA detector. The mobile phase consisted of a phosphate buffer (100 mM) with TBHS (10 mM) and methanol [water:methanol; 60:40% (v:v)] at 0.4 mL·min-1 flow rate. Gradient elution was applied, and all metabolites were separated in 30 min, where water: methanol percentage was raised from zero to 90%. PCr and nucleotides were simultaneously detected at 210 nm and 254 nm, respectively. ATP and PCr levels were normalized per mg of cell protein.

##### 2.4.3.2 Quantitative Analysis of Oxidative Phosphorylation and Glycolysis Contribution to Total ATP Production

To estimate ATP production through mitochondrial respiration and glycolysis, we measured ATP/ADP ratios in the presence of OXPHOS (rotenone, antimycin A and oligomycin 1 μg/mL) or glycolysis (2-deoxyglucose) inhibitors, as described previously (27). As 2-deoxyglucose is a competitive inhibitor, it was used at equimolar concentrations with medium glucose levels for MCF7, MDA-MB-231, and MCF10A.

##### 2.4.3.3 Phosphometabolite Isolation

Cellular phophometabolites (i.e. ATP and PCr) were isolated by LC (GE Healthcare ÄKTAPrime Plus) using a Mono Q HR 5/5 ion-exchange column (Pharmacia Biotech) with triethylammonium bicarbonate (TEAB) buffer pH 8.8 (gradient from 0-85%) at a 0.4 ml min-1 flow-rate, equipped with a UV detector fixed at 280 nm. All phosphometabolite fractions were collected by LC using this method, unless stated otherwise. Each sample was divided into two fractions, PCr and ATP, which were stored at -80 and -20°C, respectively.

Enzymatic processing reactions were used to transfer phosphoryl groups from ATP and PCr to glycerol. The γ-phosphoryl of ATP was transferred to glycerol by glycerol kinase, and β-phosphoryls of ATP was transferred to glycerol by coupled catalytic reactions of adenylate kinase and glycerol kinase. The phosphoryl group of PCr was transferred to glycerol by combined catalytic reactions of creatine kinase and glycerol kinase. Particular procedures in further detail:

ATP (γ-phosphoryl of ATP) fractions were lyophilized and reconstituted with a 200 µL mixture of ultrapure water, 10mM TEAB (pH 8.8), 2mM MgCl_2_, 5mM glycerol, and 1 µL of glycerol kinase. The mixture was incubated at 37 °C for 1 hour, and fractions of G3P (γ-phosphoryl of ATP) and ADP (β-phosphoryl of ATP) were collected by LC.

ADP (β-phosphoryl of ATP) fractions were lyophilized and reconstituted with 200 µL mixture of ultrapure water, 10mM TEAB (pH 8.8), 2mM MgCl_2_, 5mM glycerol, 1 µL of glycerol kinase, and 1 µL of adenylate kinase (myokinase). The mixture was incubated at 37°C for 2 hours, and the fraction of G3P (β-phosphoryl of ATP) was collected by LC.

The phosphoryl group from PCr was transferred to G3P in a two-stage process:

The phosphoryl from PCr was transferred to ADP. PCr fraction of a sample was lyophilized and reconstituted in 200 µL mixture of ultrapure water, 25mM TEAB (pH 8.8), 1mM MgCl_2_, 200µM ADP, 20µM diadenosine pentaphosphate, 1mM dithiothreitol, and 500 µg/mL creatine kinase. The mixture was incubated 37°C for 2 hours, and fractions of ATP were collected by LC.

The phosphoryl from ATP was transferred to glycerol. ATP fractions were freeze-dried/lyophilized and reconstituted in 200 µL mixture of ultrapure water, 10mM TEAB (pH 8.8), 2mM MgCl_2_, 5mM glycerol, and 1 µL of glycerol kinase. The mixture was incubated at 37°C for 1 hour, and fractions of G3P (phosphoryl of PCr) were collected by LC.

#### 2.4.4 Stage IV: Analysis of ^18^O Isotope Distribution

Distinct samples containing phosphoryls of γ-ATP, β-ATP, PCr enzymatically transferred to G3P and G6P, G3P (extracted by methanol-water) were converted to respective trimethylsilyl derivatives with Tri-Sil as the derivatization agent. ^18^O incorporation rate into phosphometabolites was determined with a GC-MS operated in select ion-monitoring mode (Agilent 7890 GC connected to an Agilent 6890 MSD, equipped with a 5 m deactivated precolumn and a 30m HP-5MS analytical column.

To perform derivatization reactions: Samples containing phosphoryls of γ-ATP β-ATP, PCr enzymatically transferred to G3P and G6P, G3P (extracted by methanol-water) were lyophilized and reconstituted with 10 µL of MOX (20 mg/mL in pyridine), vortexed thoroughly for 15 seconds and incubated at 30°C for 90 min. 40 µL of MSTFA+1%TMCS was added, vortexed for 15 seconds, and incubated at 37°C for 30 min. Samples were cooled to room temperature for 5 minutes, centrifuged at 12000 g for 3 minutes, and transferred (supernatant) to vials (200 µL inserts) for GC-MS analysis.

The instrument was operated in split injection mode with a temperature gradient of 60°C to 325°C with the rate of 10°C/min, hold time 10 min. Injection volume was 5 µL, split ratio 10:1, inlet temperature 250°C and flow rate (He) 1.1 mL/min in scan mode: For G3P ions were selected at 357, 359, 361, and 363 m/z and for G6P were selected at 387, 389, 391 and 393 m/z, which represent the isotopes of ^16^O replaced with ^18^O at one, two or three positions in the phosphoryl group. Isotope abundances were derived from integrals of their peak areas, which correspond to the fractions of the replaced oxygens.

The cumulative percentage of phosphoryl oxygens replaced by ^18^O in the metabolites was calculated using the formula:

[%O118+2(%O218)+3(%O318)+…+n(%On18)/[n(%O18inH2O)],

where n is the total number of phosphoryl oxygen sites in the metabolite

### 2.5 Bioinformatics Analysis

AK2 and MtCK1A gene analysis of primary HBC was based on the Cancer Genome Atlas (TCGA) platform (http://www.cbioportal.org/) where the Pan-Cancer Atlas database was used. Further information on parameters and conclusions is available in **Figures 4**, **5**.

To perform Kaplan–Meier (K–M) analysis on AK2 and AK6 gene we used an open access online survival analysis tool (28). We primarily analyzed data from Lymph node positive and TNBC patients. For breast cancer patients’ hazard-ratios were calculated using best auto-selected cutoff. K–M curves were created using the online K–M plotter (https://kmplot.com/analysis/index.php?p=service&cancer=breast). The latest 2021 version of the database was utilized for all these analyses.

### 2.6 Statistical Analysis

Data are expressed as mean ± SEM of taken over ≥3 independent experiments, ≥3 technical replicates per experiment. Differences between experimental groups were determined by ANOVA followed by Holm-Sidak tests and for K-M analysis Log-rank test was performed. A p value of less than 0.05 was considered significant.

## 3 Results

### 3.1 Bioenergetic Status of HBC Cells: Increased Intracellular ATP Level, Reduced ATP Turnover and Shift of ATP Production From OXPHOS to Glycolysis

**Figure 2:**
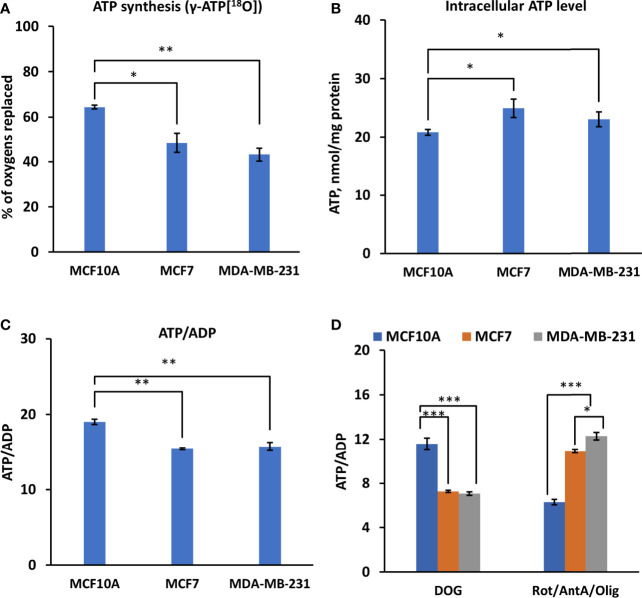
Assessment of γ-ATP[^18^O] turnover and bioenergetic profile in Luminal A, Triple-negative breast cancer cells, and control cells. Comparative analysis of intracellular **(A)** ^18^O-metabolic labeling of γ-ATP, reflecting ATP synthesis *via* OXPHOS. **(B)** Changes in ATP level and **(C)** ATP/ADP ratio between Luminal A (MCF7), Triple-negative (MDA-MB-231) breast cancer, and control cells (MCF10A). **(D)** Effect of glycolysis and OXPHOS inhibition on the intracellular ATP/ADP ratio. All data presented as mean ± SEM (ANOVA followed by Holm-Sidak test n=3-5). *, ** and *** indicate a statistically significant differences between the mean values; p < 0.05,p < 0.01 and p < 0.001, respectively. AntA – antimycin A, DOG – 2-deoxyglucose, Olig – oligomycin, Rot – rotenone.

Unlike in normal cells, hybrid metabolic state, where glycolysis and OXPHOS are simultaneously active, was observed in TNBC cells (5). Hence, the bioenergetic status of HBC cells was evaluated by measuring the static intracellular ATP levels and ATP/ADP ratios. Moreover, to assess the dynamics of energy fluxes *via* OXPHOS, we applied ^18^O-labeling of ATP by labeling of γ-ATP[^18^O]. Labeling of γ-ATP[^18^O] predominantly occurs in mitochondria by phosphorylating ADP with inorganic phosphate Pi[^18^O] derived from cellular ATPase reactions (ATP + H_2_[^18^O] → ADP + Pi[^18^O]). Measurements revealed a significant reduction in the ^18^O-labeling of γ-ATP of MCF7 and MDA-MB-231 cell lines compared to control cells (**Figure 2A**), indicating a decrease in OXPHOS activity. Diminished γ-ATP[^18^O] turnover is a new feature of breast cancer cell metabolism, reflecting intrinsic rearrangements in energetic networks.

Analysis of intracellular ATP levels revealed an increase in ATP content in both cancer cell lines (**Figure 2B**). A slightly diminished ATP/ADP ratio was observed in both HBC cell lines (**Figure 2C**), indicating a different energy state. The contribution of glycolysis or OXPHOS into energy metabolism was assessed using the respective inhibitors and by observing changes in the ATP/ADP ratio. We observed apparent differences between cancer and control cells. A substantial decrease in the ATP/ADP ratio in the presence of a glycolysis inhibitor (2-deoxyglucose) in MFC7 and MDA-MB-231 cells, compared to MCF10A, suggests that glycolysis plays a significant role in energy production in HBC cells (**Figure 2D**). At the same time, inhibition of OXPHOS (with oligomycin, rotenone, and antimycin A) had the weakest effect on the ATP/ADP ratio in MDA-MB-231 cells and the strongest in the MCF10A cells (**Figure 2D**). Based on the pattern of changes in ATP/ADP ratios, we conclude that in MCF7 and MDA-MB-231 cells, most ATP (70%) was produced *via* the glycolytic pathway, indicating a metabolic shift compared to MCF10A cells, where the main provider of ATP was OXPHOS (**Figure 2D**).

### 3.2 Glycolytic Flux, HK Expression Pattern in MDA-MB-231 Cells, and Mitochondrial Substrate Shuttle as a Link Between OXPHOS and Glycolysis in MCF7 Cells

**Figure 3:**
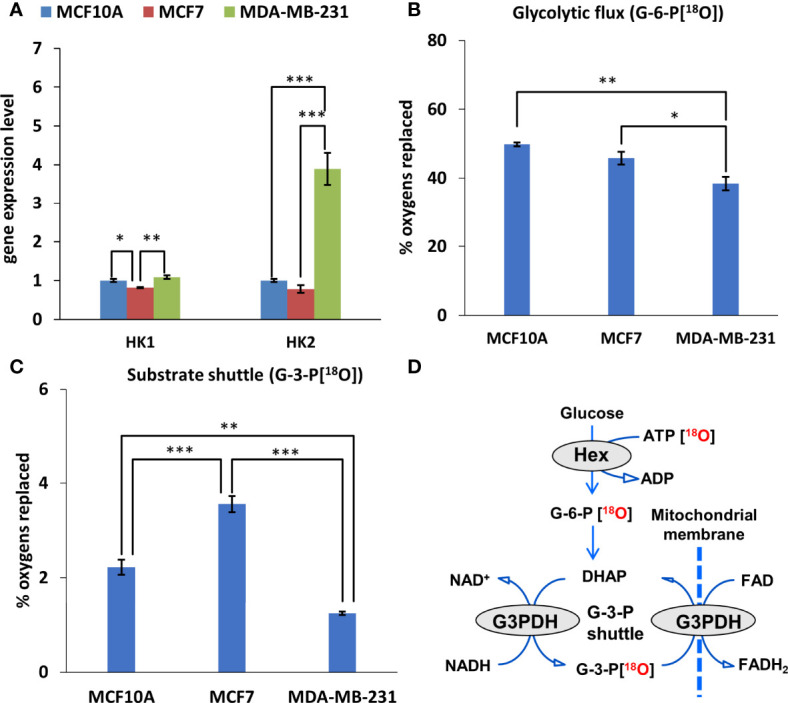
Glycolytic and mitochondrial substrate shuttle profile of Luminal A, Triple-negative breast cancer cells, and breast control cells. **(A)** HK (fold change of the mRNA level) isoform expression pattern in Luminal A (MCF7), Triple-negative (MDA-MB-231) breast cancer cells, and breast control cells (MCF10A). ^18^O-metabolic labeling of **(B)** G-6-P[^18^O] and **(C)** G-3-P[^18^O] reflecting glycolytic and substrate shuttle activities, respectively. **(D)** Schematic representation of metabolite ^18^O-labeling allowing for tracking glycolytic and G-3-P substrate shuttle dynamics. All data are presented as mean ± SEM (ANOVA followed by Holm-Sidak test n=3). *, ** and *** indicate statistically significant difference between the mean values, p < 0.05, p < 0.01, and p < 0.001, respectively. HK – hexokinase; G-6-P – glucose-6-phosphate; G-3-P – glycerol-3-phosphate.

As we observed an increased contribution of glycolytic pathway to energy production in breast cancer cells compared to control cells. Therefore, we evaluated the HK expression pattern in those cells. **Figure 2A** illustrated that TNBC cells (MDA-MB-231) had four times higher HK2 mRNA expression levels than had Luminal A (MCF7) and normal breast epithelial cells (MCF10A). Nevertheless, no difference in HK1 expression level was observed between MDA-MB-231 cells and control cell lines (MCF10A cells) (**Figure 3A**). For MCF7 cells, even slightly lower HK1 expression level was observed compare to MDA-MB-231 cells (**Figure 3A**).

Next, we estimated how distinct HK pattern (**Figure 3A**) and increased glycolytic activity (**Figure 2D**) of breast cancer cells is related with glycolytic flux. Observation of the ^18^O-labeling of G-6-P[^18^O], which reflects the glucose consumption rate of energy metabolism, does not reveal any difference between MCF10 and MCF7 cells (**Figures 3B, D**).

The glycolytic flux, however, was significantly lower in MDA-MB-231 cells compared to MCF10 cells. At the same time, inhibition of glycolysis in MDA-MB-231 cells had a similar suppressive effect on the ATP/ADP ratio as in MCF7 cells (**Figure 2D**), indicating nearly equal activity of glycolysis in these cells. This discrepancy could be due to the lower turnover and labeling of γ-ATP[^18^O], a precursor for G-6-P[^18^O]. Yet, this was not the case in MCF7 cells, which also had lower γ-ATP[^18^O]. Moreover, we did not observe correlation between HK level and G6P flux in MDA-MB-231 cells (**Figures 3A, B**).

Metabolic flux through the G-3-P shuttle, which reflects the mitochondrial substrate shuttle (**Figure 3D**), is an important supplier of reducing equivalents to mitochondria, especially for MCF7 cells. We observed that the MCF7 cell line is clearly distinguished by its high levels of labeled G-3-P[^18^O] (**Figure 3C**). Since HK2 expression in MCF7 cells was low, it can be assumed that the interaction between OXPHOS and glycolysis is realized *via* the G-3-P shuttle in these cells.

### 3.3 A Decreased CK Metabolic Flux and a Lower PCr Level Is Associated With Downregulation of Mitochondrial CKMT1A in Breast Cancer Cells

**Figure 4:**
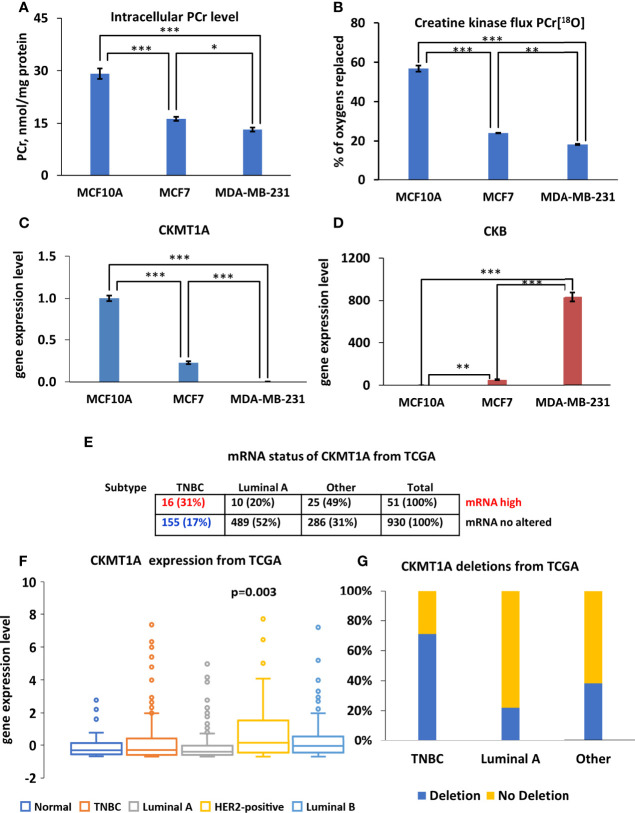
Characterization of creatine kinase (CK) network in Luminal A, Triple-negative breast cancer (TNBC) cells and breast control cells. **(A)** Comparative analysis of intracellular PCr levels in Luminal A (MCF7), Triple-negative (MDA-MB-231) breast cancer and breast control cells (MCF10A). **(B)** ^18^O-metabolic labeling of PCr[^18^O] reflecting CK flux. Expression pattern of **(C)** CKMT1A (fold change of the mRNA level) and **(D)** CKB (fold change of the mRNA level). **(A–D)** Data are presented as mean ± SEM (ANOVA followed by Holm-Sidak test n=3-5). *, ** and *** indicate a statistical significant difference between the mean values; p < 0.05, p < 0.01 and p < 0.001, respectively. The Cancer Genome Atlas (TCGA) database was utilized to analyze CKMT1A **(E)** mRNA status **(F)** expression patterns (ANOVA followed by Holm-Sidak test) and **(G)** CKMT1A gene deletion patterns in different breast cancer subtypes. PCr – phosphocreatine.

Recently, Kurmi et al. reported that the PCr energy shuttle has an important role in maintaining breast cancer cell homeostasis (20). In current work, diminished energy flux of PCr was observed in both Luminal A and TNBC cells compare to normal breast epithelia cells (**Figures 4A, B**). Labeling of PCr[^18^O] revealed approximately three-fold lower CK metabolic flux in MCF7 and MDA-MB-231 cells than in MCF10A cells (**Figure 4B**). Also, MCF7 and MDA-MB-231 cells had two-fold lower intracellular PCr levels compared to control cells (**Figure 4A**). An analysis of CKB and CKMT1A genes was conducted to evaluate how the decreased fluxes are associated with changes in CK gene expression. Analysis of mRNA level for all examined groups (**Figures 4C, D**) revealed that in both cancer cell lines CKMT1A was downregulated compared to MCF10A cells (**Figure 4C**). In contrast, mRNA levels of cytosolic CK-B were significantly overexpressed in both cancer cell lines (50- and 800-fold in MCF7 and MDA-MB-231, respectively) (**Figure 4D**). In addition, analysis of the TCGA database revealed that CKMT1A mRNA levels are elevated only in 5% of HBC patients. Furthermore, an elevation of CKMT1A expression levels was associated with amplification of CKMT1A copy number (**Figure S1**). Out of high MTCK1A level, 31% belong to the TNBC group and 20% of patients to Luminal A group, whereas most (49%) belong to Luminal B and HER2-positive groups (**Figure 4E**). Nevertheless, the only statistically significant difference in CKMT1A expression was observed between the primary HER2-positive breast cancer subtype and normal tissue (**Figure 4F** and **Figure S2**). According to the TCGA database, CKMT1A is deleted in 40% of breast cancer patients (**Figure 4G**), most of them are belonging to TNBC and Luminal A groups (71% and 22%, respectively). Altogether, this data indicates that PCr circuit is disrupted and displays low activity in Luminal A and TNBC cells, probably due to downregulation of CKMT1A.

### 3.4 Decreased AK Flux and AK2 and AK6 Isoform Overexpression in MDA-MB-231 Cells

**Figure 5:**
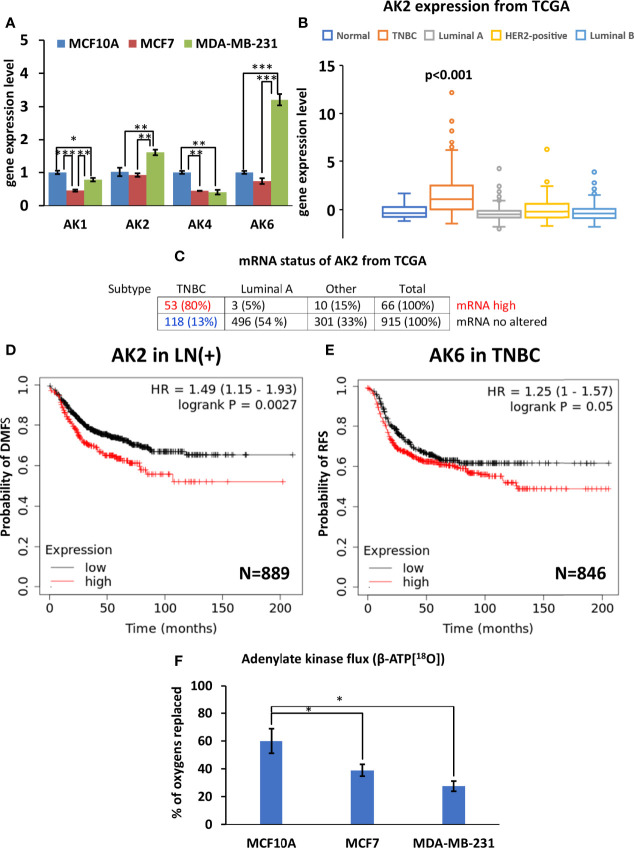
Characterization of adenylate kinase (AK) network in Luminal A, Triple-negative breast cancer (TNBC) cells, and breast control cells. **(A)** Expression patterns of AK (fold change of the mRNA level) isoforms in Luminal A (MCF7), Triple-negative (MDA-MB-231) breast cancer, and control cells (MCF10A). The Cancer Genome Atlas (TCGA) database was utilized to analyze **(B)** AK2 expression patterns (ANOVA followed by Holm-Sidak test) and **(C)** mRNA status in different breast cancer subtypes. Kaplan-Meier survival analysis showing the relationship between **(D)** AK2 mRNA expression and distant metastasis-free survival (DMFS) in lymph node-positive (LN(+)) breast cancer patients, and the relationship between **(E)** AK6 mRNA expression and recurrence-free survival (RFS) in TNBC patients. **(F)** ^18^O-metabolic labeling of β-ATP[^18^O], reflecting AK flux. **(D, E)** Statistical analysis was performed using the Log-rank test. **(A, F)** are presented as mean ± SEM (ANOVA followed by Holm-Sidak test n=3-5). *, ** and *** indicate statistically significant differences between the mean values; p < 0.05,p < 0.01, and p < 0.001, respectively.

Analysis of the four AK genes (AK1, AK2, AK4, and AK6) mRNA expression in breast cancer cells revealed several alterations in the composition of the AK network. Reprogramming of the AK network in MDA-MB-231 cells (**Figure 5A**) is associated with the upregulation of AK2 and the nuclear/cytosolic AK6 isoforms. At the same time, expression of the cytosolic AK1 isoform and mitochondrial AK4 were lower in MCF7 and MDA-MB-231, compared to MCF10A (control). Analysis of the TCGA database revealed that the AK2 mRNA level is elevated only among 6% of HBC patients. Yet, most patients with high AK2 mRNA levels are in the TNBC group (80%), while only 5% are in the Luminal A group (**Figure 5C**). To strengthen results statistical analysis was performed. Only in AK2 gene expression statistical differences were found between TNBC and normal breast tissue (**Figure 5B** and **Figure S3**). In addition, expression of AK6 and AK2 at the protein level in all cell lines studied was demonstrated by western blot (**Figure S4**). We did not noticed correlation between protein level and Ak2 and Ak6 gene expression level (**Figure S4**). To evaluate the clinical significance of AK2 and AK6, we used K-M survival analysis (**Figures 5D, E**). Interestingly, high AK2 levels of tumors were associated with poor outcome of distant metastasis-free survival in breast cancer patients with positive lymph node compared to those tumors which have low level of AK2 (**Figure 5D**). Moreover, we found that AK6 affected recurrence-free survival among TNBC patient (**Figure 5E**). Survival analysis showed that TNBC patients with high AK6 have poor outcome compared to TNBC patients with low level of AK6 (**Figure 5E**). These results indicate that AK2 and AK6 gene expression could have considerable clinical significance in diagnosing aggressive breast cancer. A unique property of AK catalysis is the ability to allow cellular utilization of the second high-energy phosphoryl of ATP (A-αP_~_βP_~_γP). ^18^O-metabolic labeling of β-ATP revealed a reduced β-ATP turnover in MCF7 and MDA-MB-231 cells, compared to control MCF10A cells. This suggests a decreased AK flux in breast cancer cells (**Figure 5F**). Altogether, this data indicates that decreased AK flux is probably associated with the downregulation of AK1 and AK4 in HBC cells. The current study revealed that both AK6 and AK2 are promising biomarkers for TNBC.

## 4 Discussion

The stable isotope (^2^H, ^13^C, ^15^N, ^18^O) tracer-based metabolomics technologies are used for metabolic research (29, 30). Water-based ^18^O-labeling has several advantages over ^13^C and ^15^N labeling. Firstly, the cellular water uptake is fast because water has a high diffusion coefficient (2.3 µm^2^/ms). Secondly, ^18^O labeling period is very short compared to the time it takes to do ^13^C or ^15^N labeling (30). Based on the Encyclopedia of Genes and Genomes (KEGG) database, approximately 3000 enzyme reactions used water as a substrate (30). In general, stable isotope-labeled water (^2^H_2_O, ${\text{H}}_{2}^{18}\text{O}$ , and H2182O ) have been used to study cellular anabolic and catabolic processes, including in cancer cells (30–32). Most important, numerous studies have shown that usage of ^18^O-enriched water is safe for humans (22).

It is well known that the backbone of cellular energy metabolism is high-energy phosphoryl turnover and dynamics of ATP synthesis and hydrolysis in OXPHOS and glycolytic pathways, and phosphoryl exchange through phosphotransfer pathways (9). However, knowledge about flexibility of phosphotransfer network in cancer cells has been limited. It is first time when ^18^O labeling method was applied to study dynamics of ATP γ- and β-phosphoryl turnover and metabolic flux through phosphotransfer pathways in cancer cells. The objective of this work was to apply the ^18^O isotope labeling technology and GC-MS-based phosphometabolite analysis to study the dynamics of the HBC energy metabolism. We directly demonstrate that compare to normal breast cells, HBC cells have a lower γ/β -ATP[^18^O] turnover rate. The lower γ-ATP[^18^O] turnover rate was associated with reduced OXPHOS activity in those cells. As a result, we observed energy metabolism shifting towards glycolysis in HBC cells. Moreover, we also monitored alterations in fluxes through HK (G-6-P[^18^O]) CK (PCr[^18^O]) and AK (β-ATP[^18^O]) phosphotransfer pathways as well as mitochondrial substrate shuttle (G-3-P[^18^O]) in HBC cells.

Resent work has demonstrated that high-ATP breast cancer cells has an aggressive phenotype with inclination to metastasis and they are multi-drug resistance, as well (33). Similarly, we observed, in both HBC cell lines, elevated intracellular ATP levels which are associated with shifting their energy metabolism towards the glycolytic pathway, compared to the control breast epithelial cell line. Other investigators have also noted that several cancer cell lines, including HBC, have increased ATP levels (34). High ATP level in cancer cells may be related with the serine/threonine protein kinase (AKT) activation. *In vitro* study has shown that AKT regulates intracellular ATP level *via* increasing activity and expression of glycolytic enzymes (35). Moreover, a higher intracellular ATP could be beneficial to cancer cells in order to keep the intracellular ATP/ADP ratio low. *In vitro* studies demonstrated that low intracellular ATP/ADP allows cancer cells to maintain a high glycolytic activity in conditions where OXPHOS activity is suppressed or altered (36). The present study demonstrated that in HBC cell lines, decline in OXPHOS and low intracellular ATP/ADP ratio enhanced glycolytic activity of cancer cells. In MCF7 cells, glycolysis was accompanied by increased flux through the G-3-P shuttle, while MDA-MB-231 cells had overexpressed HK2 instead. In current study, we did not observe a correlation between HK2 expression and G-6-P[^18^O] fluxes for MDA-MB-231 cells. It is known that enzyme expression level or activity sometimes is different from metabolic flux. Enzyme activity represents the upper metabolic limit, yet actual flux depends on current metabolic needs. For example, in the heart muscle with high AK and CK activities, ^18^O-measured metabolic flux represents only a fraction of total activity (37). Moreover, in failing hearts, the total activity of adenylate kinase is decreased, yet metabolic flux is increased. Metabolic fluxes catalyzed by adenylate kinase, creatine kinase, and hexokinase correlate with heart functional load at the same level of total activity (38). Another possible explanation could be the different intracellular localization of HK2 in studied cells. It has been demonstrated that HK2 binding to mitochondria facilitates the G-6-P channeling into glycolysis in cancer cells. At the same time, when HK2 is localized in the cytosol, the G-6-P is directed into the pentose phosphate shunt (16). Further studies are needed to clarify how HK expression pattern and localization influence the glycolytic flux in breast cancer cells.

Metabolic reprogramming in breast tumors usually depends on HBC molecular subtypes. It was recently reported, that the PCr energy shuttle has an important role in maintaining cell homeostasis in HER2+ positive HBC (20). Our study shows that the suppression of PCr energy shuttle in MCF7, and MDA-MB-231 cells, is associated with CKMT1A downregulation in both cell lines. Diminishing of CKMT1 is probably not specific for TNBC and Luminal A alone but is also common for other cancers like colorectal cancer (39), neuroblastoma (13), prostate cancer (40), and sarcomas (41). Recent studies have shown that inhibition of CKMT1 decreased intracellular ATP levels in leukemia and HER2+ type of HBC (20, 42). However, in the current study, no such reduction in ATP level were noticed in breast cancer cells. On the contrary, we observed increased intracellular ATP levels in MDA-MB-231 and MCF7 compared to MCF10A, normal epithelial cells. The absence of CKMT1A could be compensated by cytosolic CK-B, which has been shown to be overexpressed in some tumors, including HBC (43, 44). In the current study, CKB was up-regulated in both MCF7 and MDA-MB-231 cells. A study on colon cancer cell line SW480 demonstrated that overexpression of CKB increased intracellular PCr levels (45). In our case, the elevation of the CKB level did not protect breast cells from diminishing of PCr levels. Since cytosolic CK functionally and structurally coupled with glycolysis (7) then overexpression/diminishing of BB-CK can alter glycolytic rate in cancer cells. It was found that knockdown of CKB cells decreased glycolysis in ovarian cancer (19). Due to this fact increased glycolysis in MCF7 and MDA-MB-231 may be associated with overexpression of CKB in those cells. Further studies are needed to understand how CK network is integrated into glycolytic network in breast cancer cells.

Our previous study showed that HBC tissue had increased AK activity compared to normal breast tissue (46). We found that in normal breast tissue, most of the total AK activity (80%) is performed by the cytosolic AK1 isoform, and the mitochondrial AK2 isoform activity is about four-fold lower. At the same time, in human HBC tissue, the proportion of AK1 decreases slightly, and the proportion of AK2 increases. The current experiment demonstrates that HBC cells have a low AK-mediated metabolic flux accompanied by downregulation of cytosolic AK1 and mitochondrial AK4 genes in both HBC cell lines. Previously, a similar effect on AK-mediated flux was observed in AK1-knockout skeletal muscle. ^18^O labeling study demonstrated that knockout of AK1 in skeletal muscles suppressed β-ATP flux (47). Another study showed that knockout of mitochondrial AK4 disrupt mitochondrial ability to maintain normal intracellular nucleotide level (48). They found that in cancer cells silencing of AK4 gene increase intracellular ATP level as well as ATP/ADP ratio *via* enhancing glycolytic flux. Similarly, in the current study, we detected high ATP levels and ATP/ADP ratios in MCF7 and MDA-MB-231 cells with the raised glycolytic rate in those cells. This demonstrates that significant type-specific changes and metabolic adjustments in the AK phosphotransfer network occur in HBC cells.

Specific differences in Ak2 and Ak6 gene expression in Luminal A and TNBC could reflect the different malignant potential of these cells. On the one hand, it has been shown that AK2 is a repressor protein of growth for breast tumor cell lines (49). On the other hand, specific overexpression of AK2 has been demonstrated in ER-negative breast tumors (50). The current study, similarly to Speers et al. work, confirmed that AK2 is up-regulated in TNBC cell line. Our analysis of AK2 mRNA status in different HBC types, based on the TCGA database, shows that HBC tumors with a high AK2 mRNA expression are likely to be of the TNBC type. Moreover, dataset analysis showed that AK2 was a prognostic marker for aggressive breast cancer with positive lymph node. Recent studies demonstrated that AK2 is overexpressed in Lung cancer cells (51) and T-cell acute lymphoblastic leukemia cells (52). For Lung cancer, the positive expression of AK2 is associated with poor prognosis of pulmonary adenocarcinoma patients (51).

In this study, we found that not only the AK2, but also the AK6 gene is up-regulated in MDA-MB-231 cells. Recently, the AK6 high expression level was found in breast cancer and colorectal cancer (53, 54). In the case of colorectal cancer, the high AK6 is also correlated with a worse patient prognosis (53). In cells, the AK6 is predominantly located in the nuclear compartment, where it is required for ribosome formation and thus promotes protein synthesis and cell growth (53). It was demonstrated that reduction of AK6 expression in cancer cells disrupts ribosome assembly and abolishes tumorigenesis of cancer cells (53). Moreover, experiments on colon cancer cell line SW480 and human testicular carcinoma NT2 revealed that AK6 gene silencing or overexpression suppressed or promoted cancer cells’ invasion potential, respectively (54, 55). Furthermore, the same studies demonstrated that knockout of AK6 induces apoptosis in malignant cells (54, 55). Therefore, AK6 could also reflect more significant malignant potential of TNBC. Interestingly, when located at the cytosolic compartment, AK6 behaves as a metabolic modulator of the cancer cells. A study on colorectal cells demonstrated that in colorectal cancer cells the AK6 can hyperactivate glycolytic metabolism by phosphorylating lactate dehydrogenase A (54). It is likely that AK6 upregulation is specific to TNBC where it supports aerobic glycolysis in malignant cells.

Recently, we have hypothesized that alteration in the AMP signaling is a key event of malignant transformation of a cell (56). Mainly, disruption of AMP signaling through AMP-activated protein kinase (AMPK) leads to reduced control over cell cycle and proliferation. The AMPK is a master regulator of anabolic and catabolic processes which activity is managed by intracellular AMP/ADP : ATP level (57). The main activator of AMPK is AK-catalyzed pathway which allosterically activates AMPK *via* increasing intracellular AMP level (58). Our current study demonstrates that breast cancer cells have low AK-mediated metabolic flux associated with diminished Ak1 and AK4 expression. A study on mice revealed that knockout of AK1 decreased AMPK activation in muscle cells (59). Recent, *in vitro* experiments on cancer cells have demonstrated that AK4 silencing increases intracellular ATP and decrease ATP/ADP ratio which leads to AMPK activation (48). Our current work and other studies (23, 24) demonstrated that ^18^O stable isotope labeling method is sensitive enough to identify specific changes in AK network at different pathological conditions, including cancer. Further studies are needed to clarify which members of AK network (AK1-AK9) possess in the most regulation of intracellular nucleotide levels and how they are associated with the activity of AMPK in TNBC.

## 5 Conclusions

In summary, the introduction of a ^18^O stable isotope-based analytical methodology to cancer research allowed us to detect subtle changes in ATP generation and energy transfer pathways in HBC cells. Altered ATP levels, decreased γ/β-ATP[^18^O], and G-6-P[^18^O] turnovers indicate a profound rearrangement of breast cancer cell energy metabolism. Increased mitochondrial substrate shuttle flux (G-3-P[^18^O]), and overexpression of HK2 suggest a tight relationship between the glycolytic phosphotransfer network and mitochondria in breast cancer cells. Alteration in the CK network is associated with decreased CK metabolic flux and lower PCr levels with downregulation of mitochondrial CK isoform CKMT1A in HBC cell lines. Despite a decreased overall AK flux in TNBC (MDA-MB-231) cells, overexpression of AK2 and AK6 was observed which could support energy metabolism in mitochondrial and nuclear microcompartments. Current work indicates that transformation of ATP generation and energy transport pathways in HBC cells is designed to support the energetic needs of specific cellular compartments (mitochondria and nuclear). Specifically, in aggressive breast cancer cells phosphoryl ^18^O isotope-labeling reveals hidden rearrangements of energy metabolism which could be modulated and targeted by therapeutic agents in the future.

## Data Availability Statement

The raw data supporting the conclusions of this article will be made available by the authors, without undue reservation.

## Author Contributions

Conceptualization, AK, PD, and TK; methodology, AK, SM, HV, and SZ; validation, AK, SM, and SZ; formal analysis, AK and SM; investigation, AK, SM, IR, MP, ER-K, and LK; resources, AT, PD, and TK; data curation, AK and SM; writing—original draft preparation, AK, MP, and PD; writing—review and editing AK, IR, MP, AT, PD, and TK; visualization, AK and SM; supervision, PD and TK; project administration, IR, PD, and TK; funding acquisition, AK, IR, PD, and TK. All authors have read and agreed to the published version of the manuscript.

## Funding

This work was supported by the Estonian Research Council grants PRG1035, PSG11, PUTJD963 and the mobility grant MOBTP51; the National Institutes of Health (R01 HL134664 and R01 HL85744) and Marriott Family Foundation; as well as Estonia national scholarship program Kristjan Jaak, which is funded and managed by Archimedes Foundation in collaboration with the Estonian Ministry of Education and Research.

## Conflict of Interest

The authors declare that the research was conducted in the absence of any commercial or financial relationships that could be construed as a potential conflict of interest.

## Publisher’s Note

All claims expressed in this article are solely those of the authors and do not necessarily represent those of their affiliated organizations, or those of the publisher, the editors and the reviewers. Any product that may be evaluated in this article, or claim that may be made by its manufacturer, is not guaranteed or endorsed by the publisher.

## Supplementary Material

Supplementary Figure 1 CKMT1A copy number amplification associated with CKMT1A mRNA expression level from TCGA database. Each dot represents individual patient samples. Two-tailed Student’s t-test two-samples unequal variance was used.

Supplementary Figure 2 Original data from TCGA database represents CKMT1A expression profile of breast cancer patients. Each dot represents CKMT1A mRNA expression level of individual patient sample.

Supplementary Figure 3 Original data from TCGA database represents AK2 expression profile of breast cancer patients. Each dot represents AK2 mRNA expression level of individual patient sample.

Supplementary Figure 4 **(A)** AK2 and **(B)** AK6 expression level in breast cancer cells detected by western blotting. Homogenized cells containing 35 µg total protein was applied. Alpha tubulin was used as a control for equal loading. Mouse kidney homogenate was applied as a positive control for AK2, whereas MCF7 cell line lysate serves as a positive control for AK6 recommended by the antibody manufacturer. To compare the size of the proteins, the position of the marker proteins with given molecular mass is shown on the left side of each panel.
