# Supplementary figures and images for: Stable Isotope Tracing Uncovers Reduced γ/β-ATP Turnover and Metabolic Flux Through Mitochondrial-Linked Phosphotransfer Circuits in Aggressive Breast Cancer Cells
Source: Front Oncol. 2022 May 31;12:892195. doi: 10.3389/fonc.2022.892195 (PMC9194814; doi:10.3389/fonc.2022.892195)

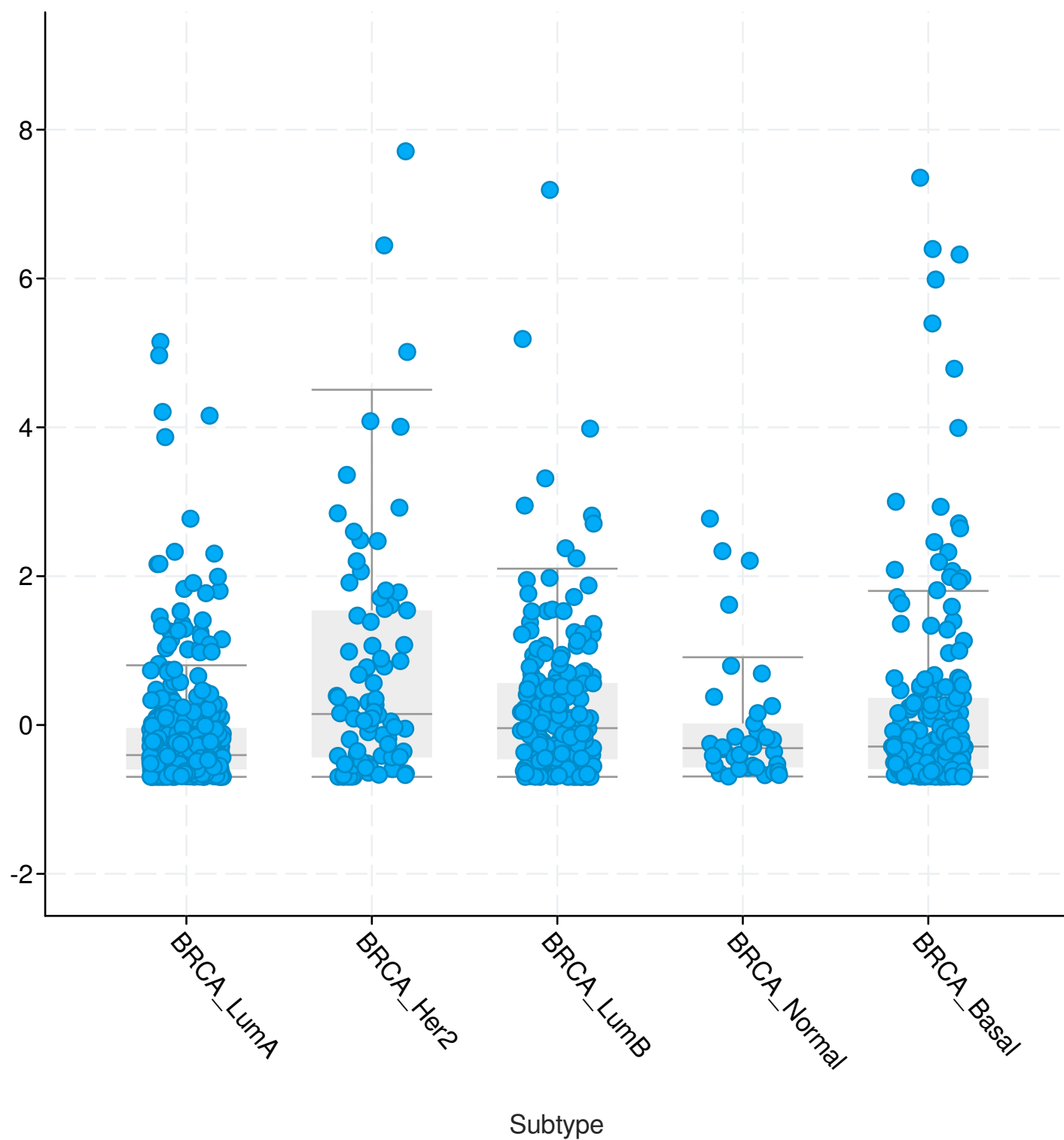

Supplement: Supplementary Figure 1 — CKMT1A copy number amplification associated with CKMT1A mRNA expression level from TCGA database. Each dot represents individual patient samples. Two-tailed Student’s t-test two-samples unequal variance was used. [file Image_1.pdf]

AK2: mRNA Expression Zscores, RSEM (Batch normalized from Illumina HiSeq\_RNASeqV2)

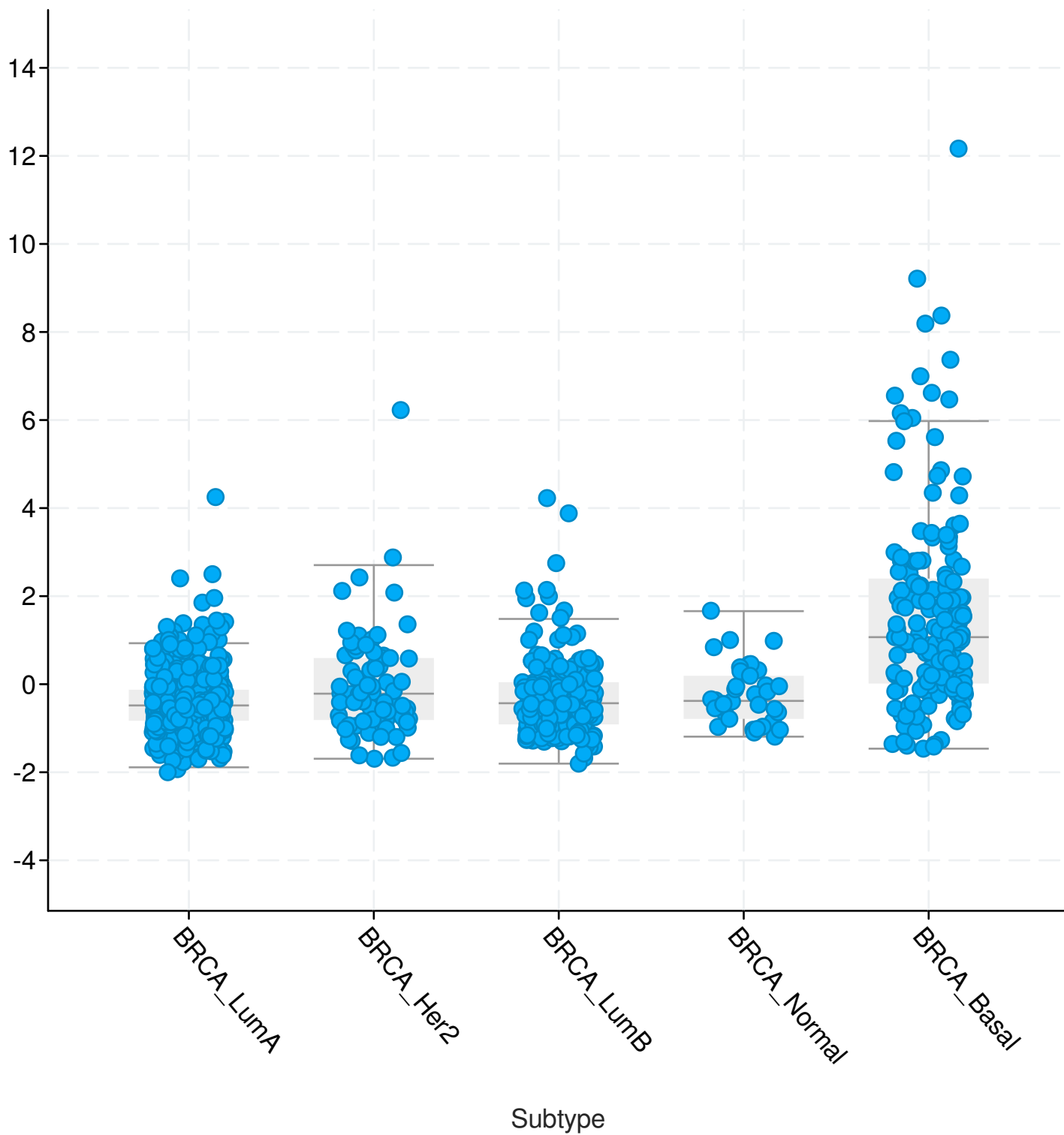

Supplement: Supplementary Figure 2 — Original data from TCGA database represents CKMT1A expression profile of breast cancer patients. Each dot represents CKMT1A mRNA expression level of individual patient sample. [file Image_2.pdf]

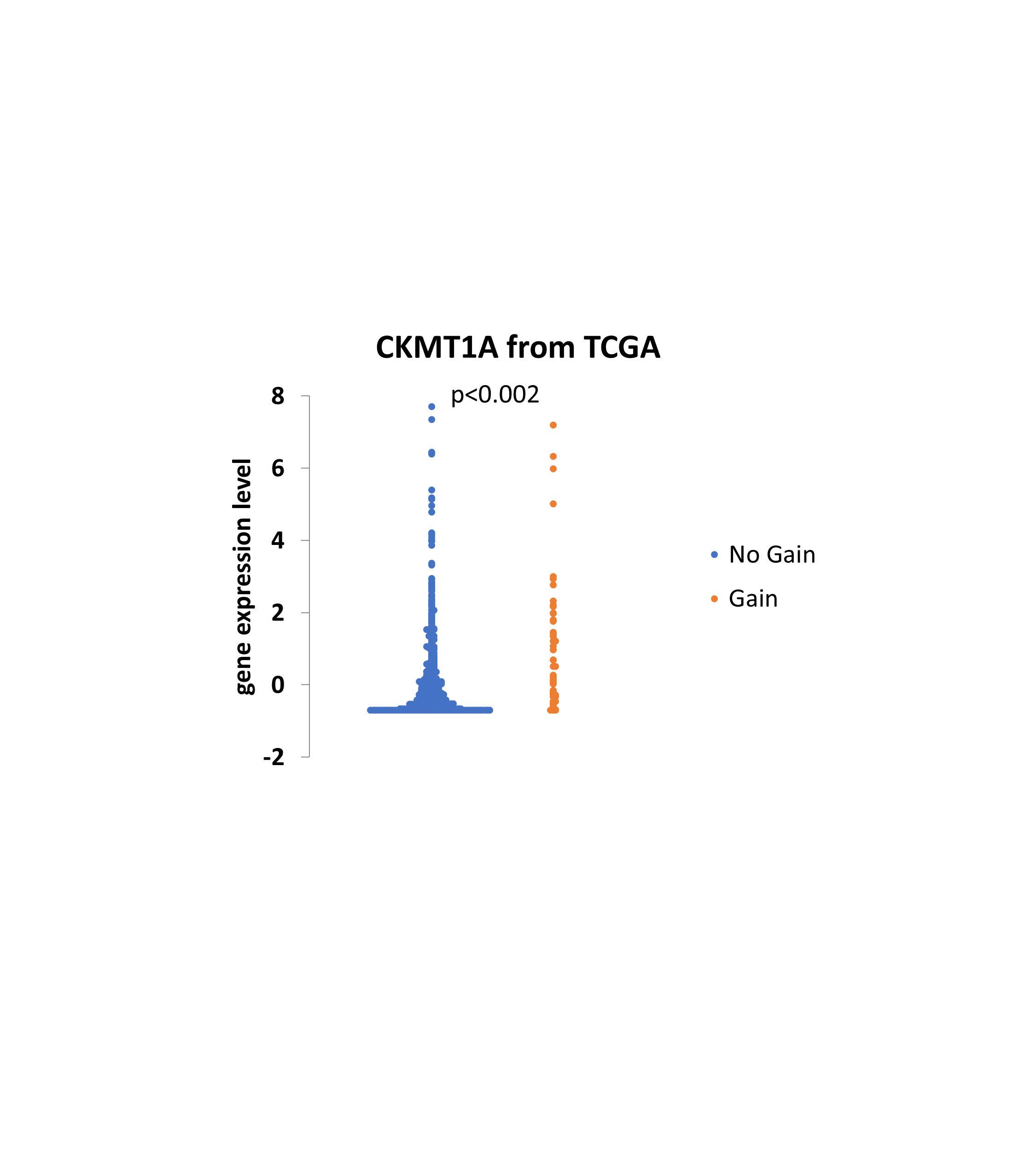

Supplement: Supplementary Figure 3 — Original data from TCGA database represents AK2 expression profile of breast cancer patients. Each dot represents AK2 mRNA expression level of individual patient sample. [file Image_3.tif]

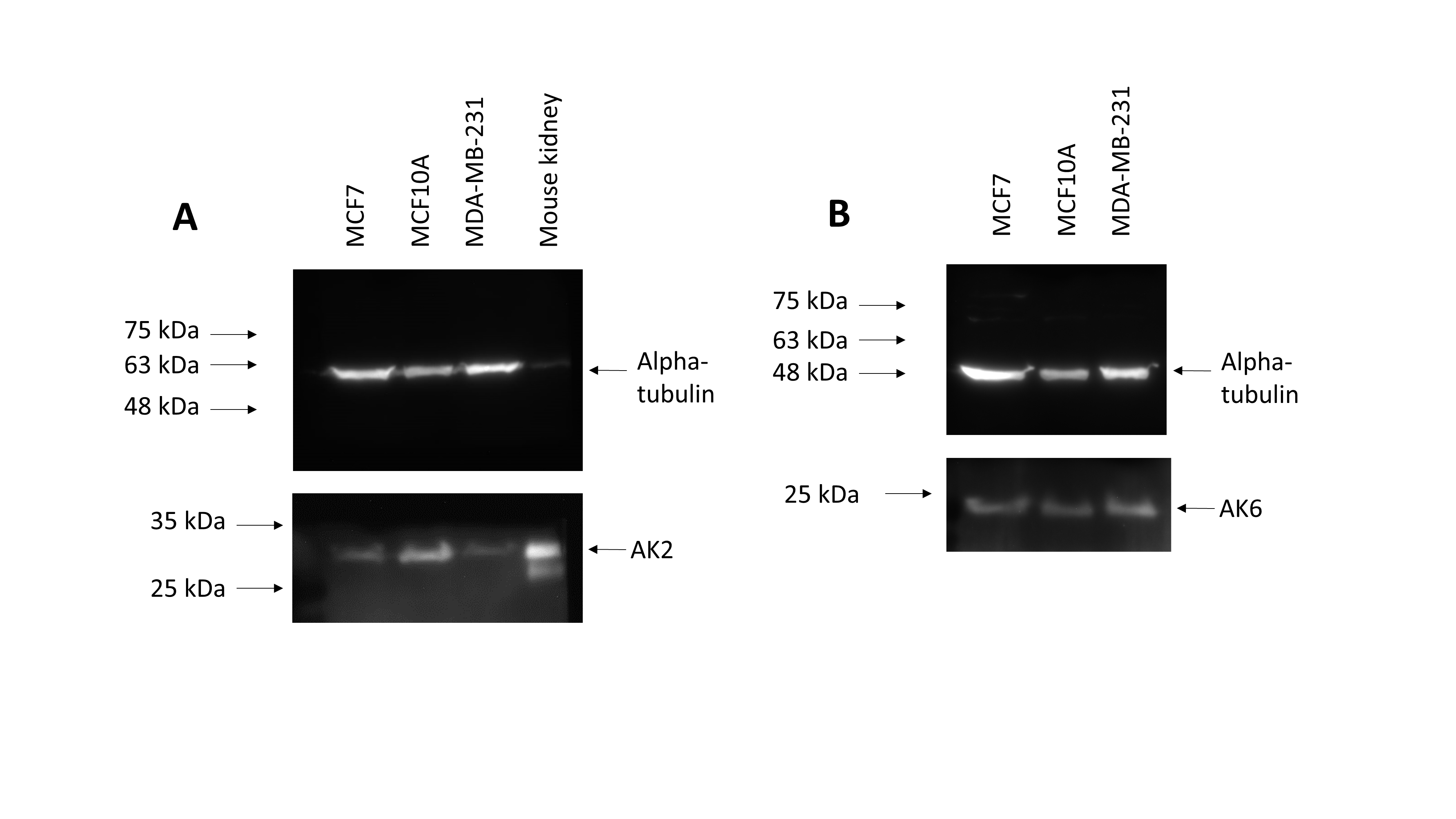

Supplement: Supplementary Figure 4 — (A) AK2 and (B) AK6 expression level in breast cancer cells detected by western blotting. Homogenized cells containing 35 µg total protein was applied. Alpha tubulin was used as a control for equal loading. Mouse kidney homogenate was applied as a positive control for AK2, whereas MCF7 cell line lysate serves as a positive control for AK6 recommended by the antibody manufacturer. To compare the size of the proteins, the position of the marker proteins with given molecular mass is shown on the left side of each panel. [file Image_4.tif]
